# Prevalence and risk factors of Hepatitis C among individuals presenting to HIV testing centers, Hawassa city, Southern Ethiopia

**DOI:** 10.1186/1756-0500-4-193

**Published:** 2011-06-15

**Authors:** Addisu Alemayehu, Yayehyirad Tassachew, Zufan Sisay, Techalew Shimelis

**Affiliations:** 1 Department of Medical Laboratory Science, Hawassa University, P. O. Box: 1560 Hawassa, Ethiopia; 2 Aklilu Lemma Institute of Pathobiology, Addis Ababa University, P. O. Box: 1176, Addis Ababa, Ethiopia

**Keywords:** Prevalence, hepatitis C virus, human immunodeficiency virus, **c**o- infection

## Abstract

**Background:**

Hepatitis C virus (HCV), either alone or in combination with Human Immunodeficiency virus (HIV), constitutes a major public health concern. This study was conducted to describe the prevalence and risk factors for HCV infection in people with and without HIV infection.

**Methods:**

Blood samples and data on socio-demographic and risk factors for HCV infection were collected from consecutive 400 HIV- positive and 400 HIV- negative individuals attending HIV testing centers in Hawassa city, from October to December, 2008. All sera were tested for antibody to HCV infection (anti-HCV) using enzyme linked immunosorbent assay (ELISA). Sera positive for anti-HCV were further tested for viral ribonucleic acid (RNA) levels using real-time polymerase chain reaction.

**Results:**

The rate of anti-HCV positivity was 10.5% in the HIV- infected individuals compared with 6% in the HIV negative group (p = 0.002). HCV-RNA was detected in 9.1% of anti-HCV positive samples and rates were comparable between HIV- infected and HIV**- **non**-**infected individuals. There was no significant difference in odds of HCV infection in participants with and without HCV risk factors in either HIV sero-group.

**Conclusion:**

HIV infected individuals had significantly higher rate of anti-HCV although most of them showed no evidence of viraemia. Hence, while priority should be given for HIV infected patients, testing those with anti-HCV for HCV-RNA remains important.

## Introduction

Hepatitis C virus (HCV) represents a major public health burden in both industrialized and developing countries. The prevalence of HCV infection is estimated to be 2.2-3.0% (130-170 million people) worldwide [1]. Region-specific estimates range from < 1.0% in Northern Europe to > 2.9% in Northern Africa, with the highest prevalence (15%-20%) reported in Egypt [2]. About 15 to 20% of HCV infections progress to potentially serious cirrhosis and end-stage liver disease [3]. Due to shared mode of transmission, co-infection of HCV and human immunodeficiency virus (HIV) has become a growing public health concern. Among the estimated 40 million individuals infected with HIV worldwide, about 4-5 million are chronically infected with HCV [4]. Prevalence and mode of transmission of each of the two viral infections are the main determinants of co-infection rate, and vary according to the geographic region. For instance, co-infection is high in patients that acquire the viruses through parenteral routes compared with those infected through sexual route [5].

In Ethiopia, a previous population-based survey had reported a moderate prevalence (2%) of HCV infection [6]. However, information is scarce regarding the rate of HIV-HCV co-infection in our setting where HIV is prevalent. This study was conducted to describe the prevalence and risk factors for HCV infection in people with and without HIV infection.

## Methods

A sero-prevalence study of HCV was conducted in people with and without HIV infection attending HIV testing centers (Hawassa Referral Hospital, Hawassa Health Centre, Organization for Social Services for AIDS (OSSA), and Beth-Zeta Hospital) from October to December, 2008. These centers are located in Hawassa, the capital city of the Southern Nations, Nationalities and Peoples' Regional State and one of the administrative regions in Ethiopia. The centers provide voluntary HIV counseling and testing services using two approaches: a client-initiated approach to serve people seeking to know their HIV status, and a provider-initiated approach which enables health care provider offer targeted medical services. Clients who are tested HIV positive are usually referred to the Hawassa Referral Hospital. Clinical and immunological assessments (CD4+ T cell count) at enrollment and at six-monthly follow-up visits identify those who are eligible for anti-retroviral therapy (ART). Those receiving ART monitor their disease status on a regular basis. Services including HIV counselling and testing, clinical and immunological assessments as well as ART are provided free of charge.

In this study, consecutive HIV-positive individuals who were at their first enrollment at the ART clinic as well as those who had been receiving ART for at least six months were recruited prospectively. Consecutive clients, who were tested HIV negative at any of the centers during the study period, were recruited for comparative purposes. The sample size was estimated using HCV prevalence rates of 4.5% and 0.8% in HIV-positive and HIV-negative subjects, respectively [7]. With 95% level of confidence and 80% power of the study, the required sample size would be 800 (400 individuals in each HIV sero-group). In both HIV sero-groups, only individuals aged 15 years and above were included. Among HIV-positive clients, those found with a CD4+ T cell counts below 50 cells/mm^3 ^were excluded from the study due to the unreliability of HCV serological tests in a state of severe immunosuppression [8].

Counsellor nurses interviewed the study subjects using structured questionnaires on socio-demographic characteristics and risk factors of HCV infection such as history of traditional and modern medical practices, sexual practices, history of sexual transmitted infections (STIs), liver disease, and family history of liver disease. Remaining sera left from HIV testing and CD4+ T cell measurement were stored at -80°C for the purpose of HCV testing. All sera were screened at the Hawassa Referral Hospital laboratory for HCV antibody (anti-HCV) using Enzyme Linked Immunosorbent Assay (ELISA) (HUMAN anti-HCV ELISA, Germany). Sera found to be positive for anti-HCV were transferred in cold boxes to the International Clinical Laboratories in Addis Ababa where testing for viral ribonucleic acid (RNA) using real-time polymerase chain reaction (PCR) (Abbott RealTi*m*e™ HCV) was performed. According to the manufacturer's guidelines, the real-time PCR test has detection sensitivity of a viral load as low as 12 IU/ml of serum. All samples were tested in accordance with the manufacturer's instructions and in duplicates to ensure reproducibility. Despite the possibility that the ELISA test introduces false positive result, we interpreted a positive anti-HCV test as HCV-exposure. Presence of HCV-RNA indicates active viral replication.

Data entry and analysis were performed using SPSS Version-16. Descriptive summary was presented in terms of mean, range, and proportions depending on the scale of the variable. Pearson's Chi-square test was used to assess differences in proportion of anti-HCV marker between HIV-infected and HIV**-**non-infected individuals. The strength of association between HCV status and risk factors was measured by calculating odds ratios. A given statistical test was reported significant whenever it resulted in a p-value < 0.05.

The study was approved by the Ethics Committees of Aklilu Lemma Institute of Pathobiology, Addis Ababa University and the College of Medicine and Health Sciences, Hawassa University. All participants gave informed written consent, and doctors managed HCV-positive cases.

## Results

Of the 402 HIV-positive and 404 HIV-negative individuals approached during the study period, 2 HIV-positive and 4 HIV-negative participants were excluded due to insufficient blood volume and refusal, respectively. The majority (94.7%) of HIV-infected participants were urban dwellers and married (48%) (Table 1). The mean age of HIV-infected participants was 31.7 years (range 18-70 years; SD 8.4) compared with 26.6 years (range 16-62 years; SD 7.3) in HIV**- **non**-**infected clients. The male to female ratio was 0.6:1 in HIV-positive and 0.9:1 in HIV-negative clients. Two-hundred (50%) HIV-infected participants were receiving ART.

**Table 1:** Socio-demographic characteristics of HIV-positive and HIV-negative individuals at Hawassa HIV testing centers, 2008

Characteristics	HIV positive (%)	HIV negative (%)	Total (%)
Residence			
Rural	21 (5.3)	17 (4.2)	38(4.8)
Urban	379 (94.7)	383(95.8)	762(95.2)
Sex			
Male	151(37.8)	191(47.8)	342 (42.8)
Female	249 (62.2)	209(52.2)	458 (57.2)
Age (years)			
<20	6(12.5)	42(87.5)	48 (6)
20-29	164(40.5)	241(59.5)	405 (50.6)
30-39	161(63.6)	92(36.4)	253 (31.6)
40-49	55(75.3)	18(24.7)	73 (9.1)
≥50	14(66.7)	7(33.3)	21 (2.6)
Marital status			
Married	192(48)	97(24.2)	289 (36.1)
Single	60(15)	274(68.5)	334 (41.8)
Divorced	60(15)	20(5.0)	80 (10 )
Widowed	88(22)	9(2.2)	97 (12.1)
Religion			
Orthodox	250(62.5)	163(40.8)	413 (51.6)
Protestant	112(28.0)	199(49.8)	311 (38.9)
Muslim	31(7.8)	37(9.2)	68 (8.5)
Other	7(1.8)	1(0.2)	8(1.0)
Educational status Educational stat			
Illiterate	57(14.2)	19(4.8)	76 (9.5)
Primary school	169(42.2)	72(18.0)	241 (30.1)
Secondary school	150(37.5)	139(34.8)	289 (36.1)
Diploma	16(4.0)	119(29.8)	135 (16.9)
Degree	8(2)	51(12.8)	59 (7.4)

The prevalence of anti-HCV in HIV-positive individuals was 10.5% compared to 6% in HIV-negative individuals (p = 0.002) (Table 2). A comparable rate of anti-HCV marker was observed in HIV-infected individuals with or without ART. HCV- RNA was detected in 6 (9.1%) of all 66 anti-HCV positive samples and viral load ranged from 31 IU/ml - 4.294 million IU/ml of serum. Rate of HCV- RNA was similar among anti-HCV- positive participants with or without HIV infection (7.1% versus 12.5%, respectively; p = 0.39), and in those HIV-infected with or without ART (5.3% versus 8.7%, respectively; p = 0.43).

**Table 2:** Distribution of anti-HCV and HCV-RNA in HIV-positive and HIV-negative individuals at Hawassa HIV testing centers, 2008

HCV Marker	Total tested	Number (%) positive	HIV-positive						HIV-negative	
			On ART		ART naives		Total			
			Tested	+ve (%)	Tested	+ve (%)	Tested	+ve (%)	Tested	+ve (%)
Anti-HCV	800	66 (8.2)	200	19 (9.5)	200	23 (11.5)	400	42 (10.5)	400	24 (6)
HCV-RNA	66	6 (9.1)	19	1 (5.3)	23	2 (8.7)	42	3 (7.1)	24	3 (12.5)

The exposure of HIV-infected and HIV**-**non**-**infected participants to different risk factors for HCV infection is summarised in Table 3. Histories of ear piercing and tooth extraction were the most frequently reported exposures in both HIV sero-groups. Among females with HIV infection, 22.5% reported having a history of abortion. Sero- prevalence rate of anti-HCV was higher in HIV-infected participants who reported having multiple sexual partners. There was no significant difference in odds of HCV infection in participants with and without HCV risk factors in either HIV sero-group (Table 3).

**Table 3:** Anti-HCV positivity in relation to HCV risk factors in HIV-positive and HIV- negative individuals at Hawassa HIV testing centers, 2008

Characteristics	HIV-positive			HIV-negative		
	
	No (%) tested	No (%) positive for anti-HCV	Crude odds ratio (95% CI)	No (%) tested	No (%) positive for anti-HCV	Crude odds ratio (95% CI)
Hospital admission						
No	300(75.0)	35(11.7)	1	342 (85.5)	21(6.1)	1
Yes	100(25.0)	7(7.0)	0.57(0.25-1.33)	58 (14.5)	3(5.2)	.83(0.24-2.89)
Blood transfusion						
No	388(97.0)	41(10.6)	1	398(99.5)	24(6.0)	
Yes	12(3.0)	1(8.3)	0.77(0.10-6.11)	2(0.5)	0	-
Unsafe injection						
No	375(93.8)	40(10.7)	1	384(96.0)	24(6.2)	
Yes	25(6.2)	2(8.0)	0.73(0.17-3.20)	16(4.0)	0	-
Multiple sexual partners *						
No	315(78.8)	31(9.8)	1	344(86.0)	18(5.2)	1
Yes	85(21.2)	11(12.9)	1.3(0.65-2.84)	56(14.0)	6(10.7)	2.1(0.82-5.74)
Sexual transmitted infections **						
No	282(70.5)	26(9.2)	1	380(95.0)	23(6.1)	1
Yes	118(29.5)	16(13.6)	1.54(0.80-3.0)	20(5.0)	1(5.0)	0.82(0.11-6.38)
Tooth extraction						
No	278(69.5)	27(9.7)	1	286(71.5)	18(6.3)	1
Yes	122(30.5)	15(12.3)	1.30(0.67-2.55)	114(28.5)	6(5.3)	0.83(0.32-2.14)
Catheterization						
No	393(98.2)	41(10.4)	1	399(99.8)	24(6.0)	-
Yes	7(1.8)	1(14.3)	1.43(0.17-12.18)	1(0.2)	0	
Scarification						
No	373(93.2)	39(10.5)	1	367(91.8)	23(6.3)	1
Yes	27(6.8)	3(11.1)	1.07(0.31-3.72)	33(8.2)	1(3.0)	0.47(0.06-3.58)
Abortion						
No	193(77.5)	18(9.3)	1	195(93.3)	11(5.6)	1
Yes	56(22.5)	7(12.5)	1.39(0.55-3.51)	14(6.7)	1(7.1)	1.29(0.15-10.8)
Ear piercing						
No	240(60.0)	22(9.2)	1	292(73.0)	16(5.5)	1
Yes	160(40.0)	20(12.5)	1.42(0.74-2.69)	108(27.0)	8(7.4)	1.38(0.57-3.32)
Surgery ***						
No	376(94.0)	40(10.6)	1	388(97.0)	24(6.2)	
Yes	24(6.0)	2(8.3)	0.76(0.17-3.37)	12(3.0)	0	-
Blood letting						
No	390(97.5)	41(10.5)	1	398(99.5)	24(6.0)	
Yes	10(2.5)	1(10.0)	0.95(0.12-7.66)	2(0.5)	0	-
Tattooing						
No	365(91.2)	38(10.4)	1	376(94.0)	23(6.1)	1
Yes	35(8.8)	4(11.4)	1.11(0.37-3.32)	24(6.0)	1(4.2)	0.67(0.09-5.16)
History of liver disease ****						
No	373(93.2)	41(11)	1	387(96.8)	23(5.9)	1
Yes	27(6.8)	1(3.7)	0.31(0.04-2.36)	13(3.2)	1(7.7)	1.32(0.16-10.6)
Contact with a person having liver disease						
No	372(93.0)	39(10.5)	1	379(94.8)	24(6.3)	
Yes	28(7.0)	3(10.7)	1.03(0.30-3.55)	21(5.2)	0	-

* having more than one sexual partner; ** have ever been diagnosed for sexual transmitted infections (excluding HIV); *** ever had minor or major surgical operation; **** have ever been diagnosed for liver disease

## Discussion

The sero-prevalence of anti-HCV was 10.5% in HIV-positive and 6% in HIV-negative individuals. None of the risk factors included in the analysis significantly increased the odds of HCV exposure. The rate of HIV-HCV co-infection in the present study was higher compared to findings in an earlier report which investigated residents of Addis Ababa (4.5%) [7]. Although we found **a **lower co-infection rate compared with developed nations [9,10], the magnitude of HCV in Ethiopia would become a concern as HIV is more prevalent in our settings.

Despite a weak association, we found a significant difference in rates of anti-HCV positivity between HIV-infected and HIV-non-infected individuals. In agreement, a previous report showed that HIV positive inhabitants in Addis Ababa had about five-fold rate of anti-HCV compared with HIV negative individuals [7]. However, associations between HCV and HIV infections in our settings were not as strong as those reported from industrialized countries where HIV can occur among HCV infected people exclusively [10,11].

Unlike most western countries, risk factors such as repeated transfusions, haemodialysis, or injection drug use are very rare in Ethiopia where transmission of HIV is primarily through heterosexual exposure [12]. While it remains to be fully established, rising evidences point to implications of sexual activity in HCV transmission [13]. Sexual transmission of HCV that might be facilitated by HIV and other STIs has also been reported [14,15]. Therefore, the role of sexual transmission should not be neglected as it may contribute to the higher rate of anti- HCV among HIV-infected individuals.

In the current study, the odds of HCV infection were not shown to be significantly affected by the exposure status of participants to various medical practices. This result contrasts with a finding from Egypt where risk factors including blood transfusion, invasive medical procedures, and frequent injections significantly increased the odds of HCV infection [16]. This may be due to the strength of risk factors varying according to HCV epidemiology in a given geographical area. Moreover, unlike a report from Brazil [13], our investigation as well as a previous study from Egypt [16] showed that the odds of HCV infection was not significantly higher in participants with tattoos or ear piercing compared with those without the respective risk factors in either HIV sero-groups.

HCV infection commonly takes achronic course and studies have reported viraemia persisting in over 80% of infected individuals [17,18]. In the present study, however, HCV-RNA was detected in 9.1% of anti-HCV positive individuals. Such a lower rate of HCV-RNA is unusual and points to a need for further investigation in our settings to confirm or challenge this finding. Factors such as intact immune status [19] and heterosexual HCV transmission [20] were suggested to contribute to varying rates of spontaneous HCV clearance in different geographical regions. In contrast, our study lacked enough power to assess whether factors such as demographic characteristics, routes of transmission, HIV and ART status were influencing rate of HCV clearance.

Findings in this study need to be interpreted in light of its methodological limitations. First, lost participants, who tested HIV positive at the centers and did not register at the ART clinic, may introduce selection bias. Second, there could be a survival bias as a result of deaths occurring among patients with viral hepatic C within the six months following the ART initiation. Third, lack of confirming ELISA positive test results using an immunoblot test might lead to misinterpretation of anti-HCV false positive results as HCV exposures.

In conclusion, HIV-infected individuals had significantly higher positivity rate of anti-HCV although most of them had no sign of viraemia. Hence, with priority given for HIV- infected patients, testing those with anti-HCV for HCV-RNA is important in order to manage HIV-HCV co-infected patients appropriately. Further studies on the epidemiology of HCV infection in our settings are imperative to plan intervention methods systematically.

## Competing interests

The authors declare that they have no competing interests.

## Authors' contributions

All authors contributed to the study design, AA carried out data collection and laboratory works; AA and TS performed data analysis and interpretation; all authors contributed to final write up and approved the manuscript.
